# Communication of Bad News to Patients: Is Honesty the Best Policy?

**DOI:** 10.6004/jadpro.2015.6.3.1

**Published:** 2015-05-01

**Authors:** Pamela Hallquist Viale

I have always strived to be honest with my oncology patients. That doesn’t mean that all patients received every piece of information at my disposal, and it doesn’t mean that I didn’t try to frame my conversations with them in the most helpful way I could imagine. We all know that delivering bad news is part and parcel of oncology care. While many of our patients ultimately survive their disease, there are so many others who do not. Caring for these patients during this phase of life involves the physical care of the patient with cancer but also the many conversations we conduct during our examinations and patient visits. Imparting knowledge to our patients about a worsening prognosis is part of what we do during the caring process; how to do it and how much information to share during our patient interactions is primarily up to the care provider. Formal training in how to deliver bad news was not a part of my professional education.

## DELIVERING BAD NEWS: INDIVIDUAL APPROACHES

In our last issue, author Mady Stovall eloquently discussed the need to bring science to the art of delivering bad news to our patients. In my years of practice, I had experiences working with several providers: All shared bad news with their patients differently. Some were very scientific and blunt; others used humor to lighten a diagnosis of metastatic disease (which didn’t always go over well); and still others were almost maternal in their approach, never overtly acknowledging that a patient was reaching the end of their journey with this fearsome disease. My own approach was to be as honest as I could while gauging the individual patient’s responses, believing that the patient was actually guiding me into revealing how much he or she really wanted to know. But when faced with a direct question regarding length of time left to live, I usually gave the scientific answer. But I added comments such as "We can’t know for sure, but statistics tell us…" or "Something can happen to any of us tomorrow, but for you, I would start doing the things most important to you so that you are ready." I might say that I didn’t expect the patient to die that week, but that getting things ready for the end of life (preparing a will, writing letters to important family members and friends) was a prudent action, and that the patient should then try to spend the time remaining doing enjoyable things. But I always felt that honest communication was the governing factor in how I approached patients at this time of their lives, believing that I would want the same if I were in that position.

## PATIENT PERCEPTION OF DELIVERY OF BAD NEWS

The "honest" approach seemed to be the most honorable and respectful thing I could do for my patients. However, a recently published paper in the Journal of the American Medical Association by Tanco et al. (2015) gave me pause. The researchers wanted to compare patients’ (N = 100) perception of physician compassion after watching videos of physicians delivering bad news, so they conducted a randomized clinical trial at an outpatient supportive care center. One video showed a physician giving a patient with advanced cancer what was essentially bad news in an optimistic fashion, while the second video showed a physician imparting a less optimistic message. The physicians were portrayed by actors who made the same number of empathetic statements while employing the same physical posture in each video. The patients then completed assessments using the Physician Compassion Questionnaire (0 = best, 50 = worst).

The results of the study demonstrated that the patients reported much higher compassion scores after watching the optimistic video approach vs. the less optimistic video (15 [range, 5–23] vs. 23 [range, 10–31]; *p* < .001). These significantly better compassion scores were also associated with a higher trust level for the medical profession: 63 patients felt the more optimistic message equated to a physician who was trustworthy as compared to the less optimistic message (Tanco et al., 2015). Out of the 100 patients, 57 preferred the physician delivering the more optimistic message, 21 revealed no preference for either physician, and 22 patients preferred the one giving the less optimistic message. The authors of the study concluded that patients preferred physicians who provided a more optimistic message. They reported that this might explain why many physicians are reluctant to impart bad news to patients, for fear of being viewed as less compassionate.

## OPTIMAL PATIENT COMMUNICATION STRATEGIES

But are we cheating our patients when we don’t give them an honest and educated answer regarding their disease and probable outcome? I agree with the authors, who state that we need further research and better educational techniques to help us communicate bad news to our patients. We want to strike a balance between communicating with the patient in the most helpful way possible, while reducing the perception of being a less compassionate provider. Perhaps the very best way to individualize our discussions with our patients is to explain at the very beginning of our interactions that difficult questions and situations may come up during their care, and ask them how they would want this type of information delivered to them.

A recently published study by Fujimori et al. (2014) examined the effects of a communication skills training (CST) program to educate oncologists regarding imparting bad news, concluding that CST is a valid approach to improve communication skills. Formalized training in improved communication strategies during patient encounters would be of help in teaching advanced practitioners optimal approaches to delivery of bad news to our patients, increase the confidence levels of providers, and patients would benefit by honest and helpful information, allowing them to make optimal decisions for themselves (Tanco et al., 2015).

How do you, as an advanced practitioner, impart bad news to your patients? Have you found CST programs to be beneficial? I welcome your comments about your experiences with this challenging but important aspect of our profession.
